# The dynamic course of spontaneous muscular contractions assessed using multiple‐point acquisition in diffusion‐weighted stimulated echo imaging

**DOI:** 10.1002/mrm.30576

**Published:** 2025-06-18

**Authors:** Martin Schwartz, Petros Martirosian, Victor Fritz, Günter Steidle, Bin Yang, Fritz Schick

**Affiliations:** ^1^ Section on Experimental Radiology, Department of Diagnostic and Interventional Radiology University of Tübingen Tübingen Germany; ^2^ Institute of Signal Processing and System Theory University of Stuttgart Stuttgart Germany

**Keywords:** diffusion‐weighted imaging, motion‐sensitizing, multiple‐point acquisition, spontaneous mechanical activities in musculature, spontaneous muscular activities, twitch contraction

## Abstract

**Purpose:**

The temporal course of spontaneous mechanical activities of musculature (SMAMs) is investigated using a novel multiple‐point diffusion‐weighted stimulated echo imaging sequence (MP‐DW‐STE) with adapted spatial and temporal resolution. For this purpose, different sequence settings and measurement parameters are applied.

**Methods:**

A single‐shot MP‐DW‐STE imaging sequence with multiple signal rephasing by small flip angle RF pulses was developed to acquire image series during spontaneous muscle contractions with duration of several hundred milliseconds. Measurements were conducted on an incoherent motion phantom and in the calf muscles of eight healthy volunteers. The number and cross‐sectional area of SMAM visualizations as well as the contractile behavior in terms of onset and duration of visible SMAMs was analyzed.

**Results:**

Measurements on the incoherent motion phantom confirmed the ability of the proposed technique to characterize dynamic incoherent motion as signal voids in series of images with a high temporal resolution of approximately 40 ms. All human subjects showed SMAMs with a median duration of 120 ms, therefore, visible in several diffusion‐weighted images in a row. Contraction time of SMAMs was in the range of 80 to 120 ms for the soleus muscle. The MP‐DW‐STE sequence settings have shown to significantly influence the mean frequency and cross‐sectional area of SMAMs.

**Conclusion:**

MP‐DW‐STE imaging allows for time‐resolved recording of spontaneous muscular contractions and provides new insights into their dynamic course. This new feature can be used for better characterization of physiological SMAMs in healthy subjects and pathological SMAMs in patients suffering from neuromuscular diseases.

## 1 INTRODUCTION

Resting human skeletal muscle of healthy subjects, and especially muscle affected by some types of neuromuscular diseases, shows spontaneous mechanical activity.^1^ Frequency of fiber twitches and affected muscular regions differ between benign and pathological conditions and can be an unequivocal sign of abnormality.^2^ Increased spontaneous muscular fasciculations in specific muscle areas represents one of the most specific symptoms in the diagnosis of some neuromuscular diseases in specific stages.^2^ Spontaneous generation of action potentials in motor neurons and following excitation of motor units (several muscle fibers connected to the axon and its terminal branches) result in muscular twitch contractions (i.e., fasciculations). In particular, in patients suffering from amyotrophic lateral sclerosis, fasciculation is an early pathophysiological feature associated with other signs of the disease, such as muscle weakness,^3^ and is, therefore, of great interest in clinical and biomedical research.

Spontaneous muscular twitch contraction is generated by the shortening of several spatially distributed muscle fibers belonging to a motor unit. This results in microscopic displacement of adjacent excited and non‐excited fibers. Hence, a 3D incoherent motion is formed. Diffusion‐weighted MRI (DW‐MRI) is inherently sensitive to incoherent motion during the dephasing and rephasing of the motion‐sensitizing gradients of the MR acquisition. Based on this, it has been shown that single‐shot diffusion‐weighted spin‐echo (DW‐SE) and stimulated‐echo (DW‐STE) imaging is able to visualize spontaneous muscular contractions as signal voids in diffusion‐weighted images (DWI), termed spontaneous mechanical activities in musculature (SMAM).^4^ These signal voids in DWI have been shown to correlate directly with small myoelectric activities detectable on the skin by surface electromyography^5^ and have a pronounced extension along the muscle fiber direction.^6^ An increase of visible SMAMs in DWI for patients suffering from amyotrophic lateral sclerosis was found for specific muscular regions.^7^, ^8^ Recording of the spontaneous activities using DW‐MRI is commonly realized by repeating the same single‐shot echo‐planar DWI sequence with a fixed repetition interval (of at least about 500 ms) resulting in asynchronous sampling of the twitch contraction. DW‐MRI in combination with electrical nerve stimulation has shown the potential to visualize the muscular twitch contraction by stimulating the motor unit with an electrical pulse and repetition of the MR acquisition with different time delays.^9^, ^10^, ^11^, ^12^, ^13^ Both techniques, with and without electrical nerve stimulation, use standard DW‐SE sequences^9^, ^10^, ^11^, ^12^, ^13^ or DW‐STE imaging^4^, ^5^, ^6^ with relatively long time delays between consecutive measurements. Without repeated electrical nerve stimulation, these DW‐MRI sequences are only able to sample one time point during a spontaneous muscular contraction. Therefore, visualization of the course of contraction over time is not feasible and, therefore, it is impossible to gain further knowledge on the temporal course of SMAMs.

STE sequences have been modified in previous works by introducing series of small flip angle refocusing pulses (instead of one 90° refocusing pulse) for high‐speed imaging^14^, ^15^, ^16^ or multislice imaging^17^, ^18^, ^19^. The use of small flip angle refocusing pulses allows the acquisition of multiple echoes (with inherently increasing diffusion weighting for longer mixing times T_M_).^20^ Further technical improvement relates to the acquisition of the full k‐space after a small flip angle refocusing pulse resulting in a multiple‐echo stimulated echo image series.^21^ Reported preliminary studies^22^, ^23^ have shown that it might be possible to visualize the muscular twitch contraction using this kind of STE adaptation, called multiple‐point in time diffusion‐weighted stimulated echo (MP‐DW‐STE) imaging. Further studies^24^, ^25^ have shown diffusion measurements using a single‐shot multi‐b‐value sequence using the same sequence concept.

The objective of this work is to explore the influence of different settings of the MP‐DW‐STE sequence for recording multiple time points of single spontaneous muscular contractions and to adapt those parameters for potential clinical applications. Furthermore, the typical temporal course of spontaneous contractions in the lower leg of healthy subjects was investigated. A newly built incoherent motion phantom was used for sequence testing.

## 2 METHODS

### 2.1 Design of the MP‐DW‐STE sequence

**FIGURE 1:**
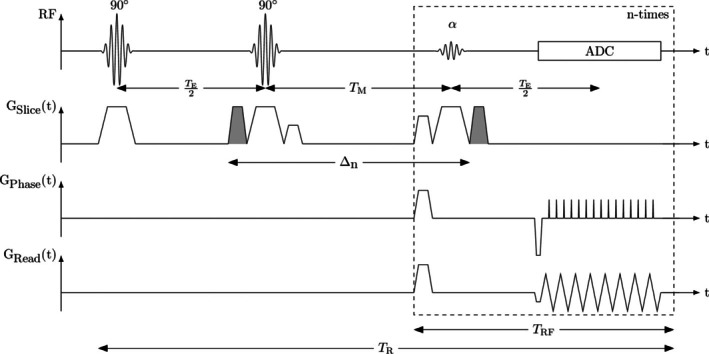
Scheme of the single‐shot multiple‐point diffusion‐weighted stimulated echo imaging sequence. The number of α‐RF pulses N_RF_, time between the α‐RF pulses (T_RF_) and mixing time (T_M_) are adjustable. Motion‐sensitizing gradient lobes are shaded in gray with the motion‐sensitizing time Δ_*n*_ of the *n*th acquisition. Motion‐sensitizing gradients were also applied as crusher gradients for selection of the stimulated echo pathway.

The sequence scheme of the single‐shot MP‐DW‐STE imaging sequence is depicted in Figure 1. The sequence was implemented with Pulseq^26^. In contrast to a conventional STE scheme, the third RF pulse after the mixing time T_M_, which deflects the stored magnetization in the longitudinal direction into the transverse plane, is replaced by a series of N_RF_ variable flip angles $\alpha_{n}=\left[\alpha_{1},\ldots ,\alpha_{N_{\mathrm{RF}}}\right],0{}^{\circ}<\alpha \leq 90{}^{\circ}\forall n\in \left\{1,\ldots ,N_{\mathrm{RF}}\right\}$ to deflect only a fraction of the stored magnetization per RF refocusing pulse. Before the signal echo is sampled, the rephasing gradient lobe for diffusion weighting is inserted. For reduction of the time T_RF_ between two subsequent α‐RF pulses, diffusion‐gradients were used as crusher gradients for selection of the stimulated echo pathway. Next, k‐space is read out by an EPI trajectory achieving the acquisition of N_RF_ single‐shot images over all N_RF_ imaging cycles.^22^, ^24^ Interference of the transverse magnetization on signal echoes in subsequent imaging cycles was suppressed by inserting spoiler gradient pulses along all three gradient directions. For signal echo readout, EPI with ramp sampling^27^ and partial Fourier was chosen to shorten the acquisition time and enable short T_RF_. Under consideration of the duration of the α‐RF pulses, crusher and spoiler gradients as well as signal readout using EPI, a temporal resolution of 40 ms was achieved on our 3 T whole body system.

Cyclic readout of the stored magnetization leads to an increase of the mixing time T_M_ in the *n*th cycle: $T_{M,n}=T_{M}+\left(n-1\right)T_{\mathrm{RF}}$. Therefore, the signal intensity in each voxel changes over the imaging cycles. This can be (at least partly) compensated by using a variable flip angle approach^18^, ^24^, ^28^ to obtain constant signal intensity for a given T_1_. With $M_{n+1}\left(t=T_{E}+T_{M}+\left(n+1\right)T_{\mathrm{RF}}\right)=M_{n}\left(t=T_{E}+T_{M}+{\mathrm{nT}}_{\mathrm{RF}}\right)$ and given the condition that the entire remaining stored magnetization should be deflected in the last cycle N_RF_ with $\alpha_{N_{\mathrm{RF}}}=90{}^{\circ}$, the flip angle series is iteratively determined by^18^, ^29^:

(1) $$\alpha_{n}=\tan^{-1}\left(e^{-\frac{T_{\mathrm{RF}}}{T_{1}}}\sin\left(\alpha_{n+1}\right)\right).$$

Increasing $T_{M,n}$ leads to an increase of the motion‐sensitizing time, and therefore, to a change of the motion‐sensitivity of each imaging cycle. The b‐value of the *n*th image acquisition without consideration of imaging gradients is

(2) $$b_{n}=\gamma^{2}G_{D}^{2}\left[\delta^{2}\left({∆}_{n}-\frac{\delta }{3}\right)+\frac{\xi^{3}}{30}-\frac{\delta \xi^{2}}{6}\right],$$

with $\Delta_{n}=\Delta_{1}+\left(n-1\right)T_{\mathrm{RF}}$; $\Delta_{1}$: initial motion‐sensitizing time of the first acquisition; ξ: rise time; δ: duration of motion‐sensitizing gradient. Therefore, the *n*th b‐value in the acquisition series depends on the initial mixing time T_M_ and the initial b‐value b_1_ following

(3) $$b_{n}=\frac{\delta^{2}\left({∆}_{1}+\left(n-1\right)T_{\mathrm{RF}}-\frac{\delta }{3}\right)+\frac{\xi^{3}}{30}-\frac{\delta \xi^{2}}{6}}{\delta^{2}\left({∆}_{1}-\frac{\delta }{3}\right)+\frac{\xi^{3}}{30}-\frac{\delta \xi^{2}}{6}}b_{1},$$

with ${∆}_{1}=T_{M}+T_{\mathrm{RF},\mathrm{Exc}.}+\delta$ and $T_{\mathrm{RF},\mathrm{Exc}.}$ being the duration of the RF pulse. The implemented MP‐DW‐STE imaging sequence will be made available for other research groups on request.

### 2.2 Incoherent motion phantom

To evaluate the DW‐MP‐STE sequence, measurements were also conducted on a custom‐built incoherent motion phantom. Because the signal voids visible in DWI result from intra‐voxel dephasing because of 3D incoherent motion, a basic implementation to reproduce this kind of incoherent motion is the induction of small local water disturbances by motion beads in a restricted area of an MR phantom filled with fluid.^6^ To increase the viscosity of the pure water and locally control the induced water disturbances, small “intra‐slice” tubes were filled with water and cellulose (2%). An overview on the structure of the phantom is given in Figure S1. Furthermore, four “inter‐slice” tubes were filled with a solution of soy‐lecithin and water to adjust different T_1_‐values mimicking the relaxation times of different tissues (T_1_: 800 ms, 1000 ms, 1300 ms, and 1600 ms).^30^, ^31^ Motion beads inside the intra‐slice tubes can be shifted by thin filaments made of polyamide and controlled by a synchronized motor unit (Arduino Due connected to servomotor devices) from the outside of the critical magnetic field (i.e., the 5 Gauss line). To prevent water exchange between intra‐slice tubes and the surrounding area, an additional layer of rubber was used to seal the intra‐slice tube for the duration of the examination. The motion bead position is, therefore, fully controllable regarding position and velocity. The microcontroller is also able to synchronize the MR imaging process by triggering the MR system using a fiber optical connection. This allows synchronizing the motion bead cycle and MR image acquisition. For testing the implemented MP‐DW‐STE sequence, a linear motion pattern was applied with synchronization of the sequence (i.e., start of the incoherent motion in the intra‐slice tube after the first DWI).

### 2.3 Study participants

Six male and two female volunteers (age: 31 ± 13 years) participated in the study after giving written informed consent. All subjects were examined in supine position. In total, 32 DW‐MRI datasets (4 different DWI measurements per subject) with summarized 74 400 DWIs were recorded. The study protocol was approved by the local ethics review board of the medical faculty of the Eberhard Karls University and the University Hospital of Tübingen in 830/2020BO2.

### 2.4 MRI acquisition settings—Healthy volunteers

All DW‐MRI measurements were conducted on a 3 T MR scanner (MAGNETOM Prisma^Fit^, Siemens Healthcare) with a 15‐channel Tx/Rx knee‐coil. In volunteer examinations, transverse slices were acquired at the position with maximum diameter of the right calf. Sequence parameters were: matrix size of 64 × 64, FOV of 192 × 192 mm^2^, slice‐thickness of 8 mm, 5/8 readout, receiver bandwidth (BW) = 2404 Hz/px, TE = 22 ms, (between consecutive STE excitations) TR = 1000 ms. A total scan‐time of 5 min per parameter set was chosen leading to a series of 300 repetitive measurements (each of them with multiple‐point imaging). Spectral fat suppression was applied. The number of RF repetitions was N_RF_ = 10 and time between two subsequent α‐RF refocusing pulses was set to T_RF_ = 40 ms. This results in a temporal resolution of 40 ms for all in vivo measurements. Flip angle series of α‐RF pulses was adjusted for skeletal muscle tissue (T_1_ = 1300 ms) following Eq. (1): *α*_*n*_ = [15.9°, 17.0°, 18.4°, 20.1°, 22.1°, 24.8°, 28.5°, 34.0°, 44.1°, 90.0°]. For investigation of the influence of the ratio between T_M_ and T_RF_ on the visible SMAMs, three different T_M_ were selected: (I) short T_M_ (29 ms) with constant increase in motion‐sensitizing time; (II) medium T_M_ (145 ms), which equals the “standard sequence settings” used in former studies^4^, ^5^; and (III) long T_M_ (189 ms). For reference (IV), a “standard” stimulated echo sequence with N_RF_ = 1, b‐value = 100 s/mm^2^ and T_M_ = 145 ms was chosen according to previous works.^4^, ^5^ All sequence parameters that are changed for the different experimental settings are given in Table 1. For anatomical reference, T_1_‐weighted fast spin‐echo (T1w‐FSE) images were recorded using the same slice positions with matrix size of 256 × 256, FOV of 192 × 192 mm^2^, slice‐thickness of 6 mm, BW of 180 Hz/px, TE = 10 ms, TR = 650 ms, and echo train length of 5.

**TABLE 1:** Sequence parameters for the four different in vivo experimental settings (I–IV) and for the phantom measurements.

			Experimental setting				
			In vivo				
Sequence parameter	Variable	Unit	I	II	III	IV	Phantom
Mixing time	T_M_	ms	29	145	189	145	145
Temporal resolution	T_RF_	ms	40	40	40	40	200
RF repetitions	N_RF_		10	10	10	1	5
Motion‐sensitizing times	Δ_1_–Δ_NRF_	ms	40, 80,…, 400	156, 196,…, 516	200, 240,…, 560	156	156, 356,…, 956
b‐Value	b_1_–b_10_	s/mm^2^	50–520	50–167	50–141	100	50–309

*Note*: b‐Values were calculated following Equation (3).

### 2.5 MRI acquisition settings—Phantom measurements

For measurements using the incoherent motion phantom, acquisition settings were adapted to N_RF_ = 5. To avoid signal voids in the entire intra‐slice motion tube, the motion bead is moved rather slowly (20 mm/s) and sequence parameters were adapted to T_M_ = 145 ms and T_RF_ = 200 ms (Table 1).

For visualization and verification of the motion‐bead position in test measurements on the incoherent motion phantom, images were acquired using a balanced steady state free precession (bSSFP) sequence with high temporal resolution (matrix size of 96 × 96, FOV of 192 × 192 mm^2^, slice‐thickness of 10 mm, BW of 1532 Hz/px, TE = 0.97 ms, and TR = 130.57 ms).

### 2.6 DW data processing and evaluation

All image reconstruction and evaluation processing steps were implemented in custom‐written code in MATLAB (The MathWorks). Intra‐series and inter‐modality spatial accordance between T1w‐FSE and DWI was achieved by an affine registration using elastix^32^, ^33^ with mutual information as similarity metric before further processing. For detection and segmentation of SMAMs in DWI, a neural network approach^8^, ^34^ was used. The used neural network architecture consists of two different sub‐networks. First a neural network based on a shallow encoder‐decoder structure inspired by U‐Net^35^, ^36^ to generate intermediate spatio‐temporal feature maps and, second, a neural network block for processing of the temporal relations between each temporal step using bidirectional convolutional long short‐term memories (CLSTMs)^37^, ^38^, ^39^. Afterward, segmentation results were revised to ensure classification results and proper subsequent evaluation. Large signal dropouts across multiple muscle regions or whole muscles were discarded since these motion patterns were assumed to be a result of intended muscular movements. Studies on the incoherent motion phantom were solely evaluated qualitatively to prove the function of the sequence. For in vivo studies, number and cross‐sectional area (CSA) of SMAMs were analyzed per repetition of the DWI measurements as well as per DWI time‐series image for each set of MR sequence parameters. SMAM activities for each N_RF_ were visualized in terms of percentage event count maps (pECMs) (i.e., the summation of SMAMs in time‐direction normalized by the number of acquired repetitions). Furthermore, temporal behavior of SMAMs was analyzed by grouping all activities regarding their onset in the DWI time‐series (i.e., the first DWI with visible signal void to the last DWI with spatial overlapping visible signal void). To analyze the dynamic contraction of SMAMs, a contraction time SMAM_TC_ was defined as the temporal period between the first occurrence in DWI to the maximum CSA of the SMAM in the DWI time‐series. For this reason, only SMAMs that were not yet visible at the first recording time in the series were evaluated, because otherwise the start time of the SMAMs would remain unclear.

### 2.7 Statistical analyses

Preliminary evaluations using a Shapiro–Wilk test have shown that the results are not normally distributed. Therefore, non‐parametric statistical tests were used for analysis. For pair‐wise statistical comparison of all three measurements settings, a non‐parametric two‐tailed Wilcoxon test was used in SPSS (IBM, version 28.0.1.1[14]). The analysis of statistical differences over all three measurement settings was performed with a non‐parametric Friedman test. In both statistical tests, a Bonferroni‐correction for detection of significant differences with a significance level of P_adj_ = 0.05 was applied.

## 3 RESULTS

### 3.1 Phantom measurements

**FIGURE 2:**
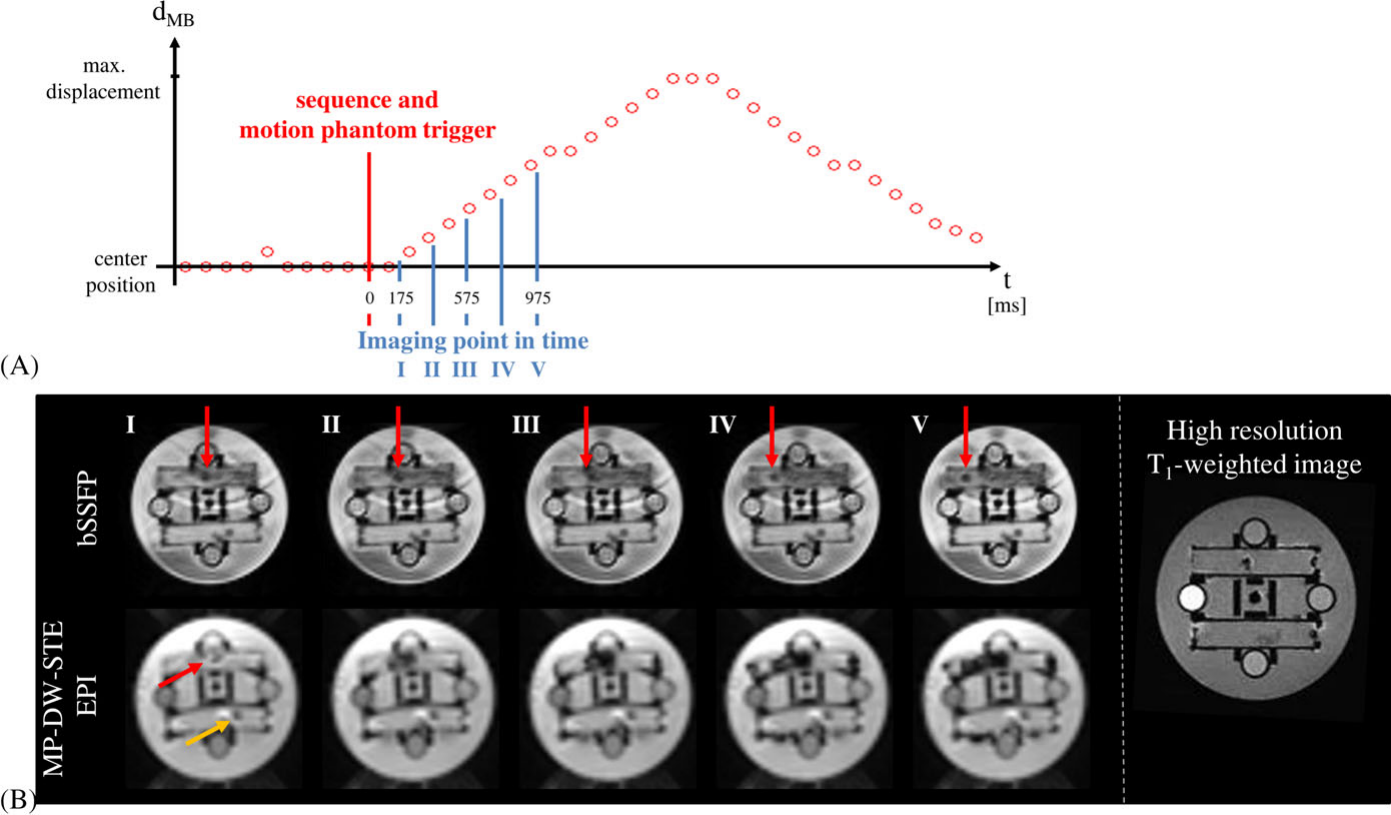
(A) Scheme of a linear motion bead displacement d_MB_ inside the incoherent motion phantom. Motion bead position represented by red circles was assessed by the short repetition balanced steady state free precession (bSSFP) sequence. Areas with signal voids in images recorded by the multiple‐point diffusion‐weighted stimulated echo (MP‐DW‐STE) sequence are depicted in blue. (B) Images of the motion bead using bSSFP (top row) and MP‐DW‐STE (bottom row) sequences. Images in the bottom row show extended signal voids resulting from incoherent motion caused by the bead displacement. The signal void propagates from the center to the left (red: moving bead, yellow: stationary bead).

To validate the implemented MP‐DW‐STE sequence, movement pattern and signal intensity within the DWI time‐series was investigated. An exemplary scheme of a linear motion pattern with sequence triggers and time points for DW imaging is depicted in Figure 2A. The signal void resulting from the water turbulence from the moving motion bead grows according to the movement visible in the bSSFP images (Figure 2B). Visualization of the dynamic motion bead movements using the MP‐DW‐STE sequence was possible in all test series (with 100 repetitions each). A maximum variation of the signal intensity over the DWI time‐series of 1.27% was measured indicating a good compensation of T_1_‐related signal intensity effects using an adjusted flip angle series following Eq. (1). Therefore, no signal interference from previous imaging cycles was assumed.

### 3.2 In vivo measurements

All measurements in healthy volunteers led to sufficient image quality for reliable analysis. However, one subject had to be excluded in further evaluation steps because of increased rate of extended signal voids indicating intended motion of the lower leg, which leads to difficulties in the evaluation of spontaneous muscular activities. An increased rate of activity in the extensor digitorum longus muscle was detected that was directly related to toe movements. This muscle did not reveal a high rate of spontaneous muscular activities in previous studies.^4^, ^5^ Variation of the rate of spontaneous activities was expected to be low during the acquisition of the four measurements, because preliminary studies have shown only a small intra‐day variability.^40^

**FIGURE 3:**
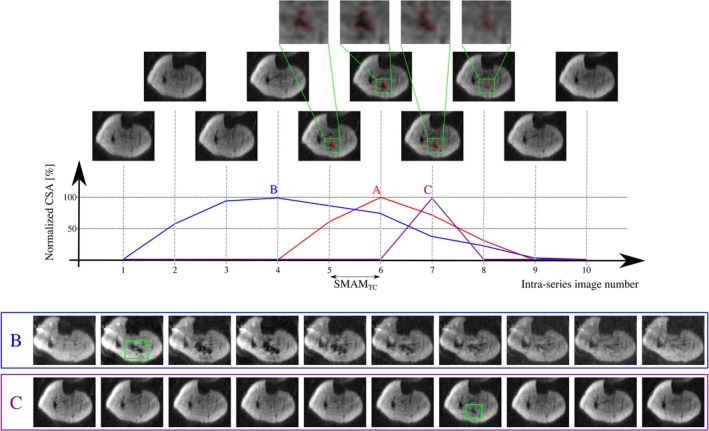
(A) Exemplary multiple‐point diffusion‐weighted stimulated echo imaging sequence (MP‐DW‐STE) image series (T_M_ = 145 ms) showing a distinct spontaneous mechanical activity of musculature (SMAM) (annotated in red) in the soleus muscle over multiple images (intra‐series image number 5–8) with corresponding normalized cross‐sectional area (CSA). An enlarged view of the SMAM is given in the upper row (highlighted in green). It can be observed that the size of the SMAM first increases and subsequently decreases. (B) Exemplary SMAM with a long temporal duration. (C) Exemplary SMAM with short temporal duration. The contraction time SMAM_TC_ is defined as temporal period between first visible signal void and maximum CSA.

A summarizing overview on the in vivo results is given in Table 2. SNR is inherently reduced compared to standard STE imaging, however, a minimum mean SNR of 26 (Table 2) was achieved, which is comparable to former studies.^4^ A mean signal reduction of minimum 49% ± 10% was found, which shows a sufficient image quality for SMAM analyses. Figure 3 shows an exemplary time‐series of DWI (T_M_ = 145 ms) with a distinct SMAM in the soleus muscle. The CSA of the SMAM reaches its maximum at the sixth time point of the image recording series. In relation to this maximum extension, the area of the SMAM in the image time‐series was: 59.5% (5th), 71.5% (7th), and 33.6% (8th). The SMAM contraction time (SMAM_TC_, given as number of imaging periods with 40 ms each) between first visible signal void and maximum CSA was quite short, as the next image after the first occurrence already shows the maximum CSA value. For comparison, a SMAM with long (Figure 3B) and short (Figure 3C) duration is depicted. The CSA of the SMAM with long duration also first increases and then decreases.

**TABLE 2:** A summarizing overview on the in vivo results in terms of image quality, the relative number of DWI indicating SMAMs and the temporal characteristic of visible SMAMs for all four experimental settings.

			Experimental setting			
			I (T_M_ = 29 ms)	II (T_M_ = 145 ms)	III (T_M_ = 189 ms)	IV (standard STE)
SNR (mean 1st image to mean 10th image)	μ		46 to 26	37 to 32	33 to 31	156
Min. mean SMAM signal drop	[%, μ ± σ]		49 ± 10	50 ± 12	52 ± 10	52 ± 10
Relative amount of DWI‐series repetitions with SMAM	Subject 1	[%]	20.0	20.0	14.0	12.3
	Subject 2	[%]	16.3	19.7	14.7	10.0
	Subject 3	[%]	3.3	5.7	6.3	3.0
	Subject 4	[%]	18.0	18.0	13.0	4.0
	Subject 5	[%]	2.0	5.0	5.0	4.0
	Subject 6	[%]	16.7	13.3	14.3	9.6
	Subject 7	[%]	22.3	14.0	15.7	10.7
	[%, μ ± σ]		14.1 ± 8.1	13.7 ± 6.2	11.9 ± 4.3	7.7 ± 3.8
Relative amount of SMAMs with:						
• Start: 1st image, end: 10th image	[%]		15.2	25.3	25.1	–
• Start: 1st image, end before 10th image	[%]		5.1	17.6	20.5	–
• Start: after 1st image, end: 10th image	[%]		73.1	55.9	53.7	–
• Start and end within time‐series	[%]		22.1	26.9	25.8	–
• Only present for two imaging points	[%]		4.2	7.5	4.2	–
Maximum SMAM_TC_	[images]		8	6	4	–
Amount of smallest possible SMAM_TC_	[%]		29.9	55.4	56.0	–
SMAM_TC_ (soleus)	Median (95% CI)		2 (2, 3) *n* = 87	1 (1, 2) *n* = 56	1 (1, 2) *n* = 50	–
SMAM_TC_ (gastrocnemius medialis)	Median (95% CI)		1 (1, 2) *n* = 28	2 (1, 2) *n* = 14	2 (1, 2) *n* = 10	–
Duration (soleus)	Median (95% CI)		3 (3, 4) *n* = 50	3 (3, 4) *n* = 49	3 (3, 4) *n* = 47	–
Duration (gastrocnemius medialis)	Median (95% CI)		3.5 (2, 5) *n* = 12	3 (3, 5) *n* = 18	4 (3, 5) *n* = 11	–

Abbreviations: CI, confidence interval; DWI, diffusion‐weighted images; Min, minimum; SMAM; spontaneous mechanical activities of musculature.

A share of 14.1% ± 8.1% of all repetitions have shown a visible SMAM in at least one DWI (experimental setup I) compared to 11.9% ± 4.3% (III) shown in Table 2. Standard STE (IV) has shown a reduced rate of repetitions showing SMAMs (7.7% ± 3.8%). A minimum number of repetitions with visible SMAMs were found for subject 5 with 2.0% (I) in contrast to 22.3% (I) in subject 7. A significant difference between I versus IV (P_adj_ = 0.04) and II versus IV (P_adj_ = 0.03) was found by analyzing the four paired measurements with a non‐parametric Wilcoxon test.

**FIGURE 4:**
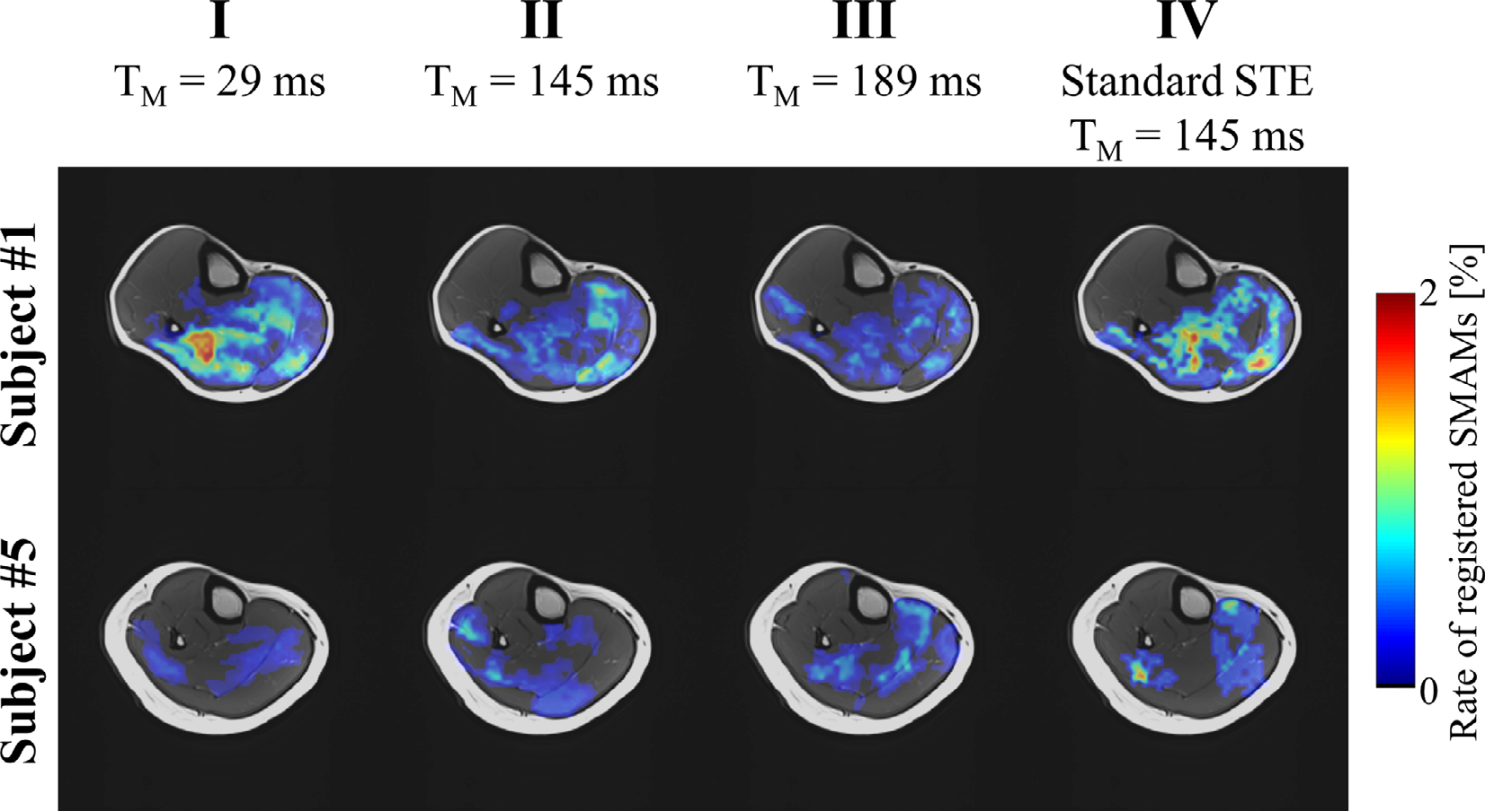
Overall percentage event count maps (pECMs) visualized for subject 1 (highest mean rate of SMAMs) and subject 5 (lowest mean rate of SMAMs) for all four MR sequence settings. It can be seen that all pECMs show the same active muscular regions. However, differences in the detected rate are clearly noticeable.

**FIGURE 5:**
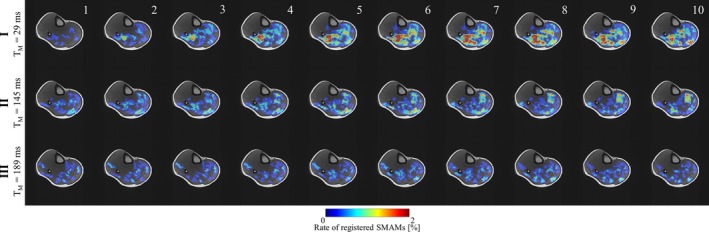
Visualization of the intra‐series percentage event count maps (pECMs) for MR sequence settings I to III for subject 1 (highest mean rate) for all 10 measurement points in time.

**FIGURE 6:**
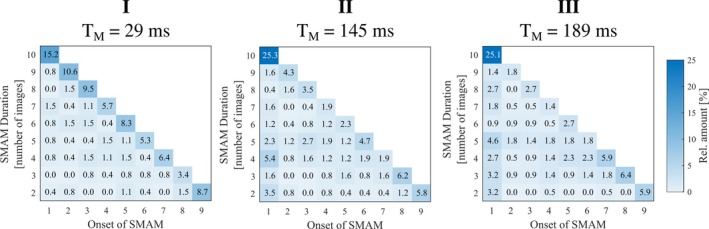
Relative distribution of the duration of visible spontaneous mechanical activities of musculature (SMAMs) (i.e., referring to the number of intra‐series images) in relation to the onset of SMAM (first imaging point in time of the diffusion‐weighted images [DWI] time‐series) for all three measurement settings. Distribution per measurement settings was normalized by the overall number of images indicating SMAMs.

**FIGURE 7:**
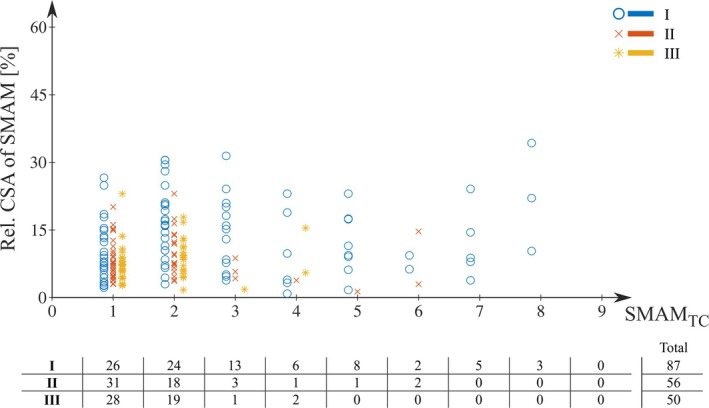
Relative maximum cross‐sectional area (CSA) of visible spontaneous mechanical activities of musculature (SMAMs) in relation to SMAM_TC_ with the number of occurrences for the soleus muscle. Relative CSA was normalized on the area of the soleus muscle per subject. SMAM_TC_ was defined as period between first visible signal void in diffusion‐weighted images (DWI) and maximum CSA.

Figure 4 indicates detection rates of SMAMs for all four MP‐DW‐STE sequence settings as pECMs for subject 1 (highest mean rate of SMAMs) and subject 5 (lowest mean rate of SMAMs). pECMs for all seven subjects are summarized in Figure S2. It is obvious that the overall patterns of active muscle regions are the same for all four settings. However, differences in the SMAM rate are noticeable. The highest rate of subject 1 in the soleus muscle is 1.94% for I, which shows a decrease to 0.81% for III. For subject 5 with a rather low amount of SMAMs, an increase is visible (0.39% [I], 0.84% [III]). pECMs for all 10 imaging points in time of DWI time‐series for subject 1 are shown in Figure 5. It can be seen that there is an increase of the activity rate from first to fifth pECM for I. III shows a distinct decrease for all 10 imaging time points. Median numbers of images per DWI time‐series time point, which indicates SMAMs are given in Table S1. An increase in the median number of images showing SMAMs from the first point in time to the maximum at seventh to ninth imaging point in time was found. The CSA of SMAMs in the unit of voxels is given for each imaging point in time. It can be seen that the spread in CSA increases for I for later time points in comparison to other experimental settings. The relative distribution of the durations of SMAMs (i.e., referring to the number of DWI time‐series images with visible SMAM at the same location) in relation to the onset of the SMAM in the DWI time‐series is given in Figure 6 for all three measurement settings. A total of 15.2% of all DWI repetitions indicating SMAMs have shown activities, which are present over the whole DWI time‐series (i.e., visible on the same spatial region from the first to the 10th DWI time‐series image) for I in contrast to 25.3% (II) and 25.1% (III). The relative shares of SMAMs with different onset and end are given in Table 2 with calculations in Figure S3A–E. The relative maximum CSA normalized by muscle area of SMAMs in DWI time‐series in relation to the SMAM_TC_ period (i.e., temporal period in number of DWI time‐series images between first visible signal void and maximum CSA) is given in Figure 7 for the soleus muscle. Interestingly, SMAMs with a prolonged SMAM_TC_ are only present for short T_M_ (I). Highest SMAM_TC_ for II was found to be 6 and 4 for III. A median SMAM_TC_ of 2 (95% CI: [2, 3]) with duration of 3 (95% CI: [3, 4]) was found for experimental setting I (Table 2) with differences to the two other experimental settings. Small differences in SMAM_TC_ and duration were revealed in the gastrocnemius medialis muscle. Other muscles were not evaluated regarding SMAM_TC_ and SMAM duration because of the low rate of SMAMs.

## 4 DISCUSSION

The incoherent motion phantom could be well used in the process of MR sequence development for assessment of the temporal course of spontaneous muscular activities. It provided a solid basis for the investigations when traversing different movement trajectories with specified speed of the motion beads.

**FIGURE 8:**
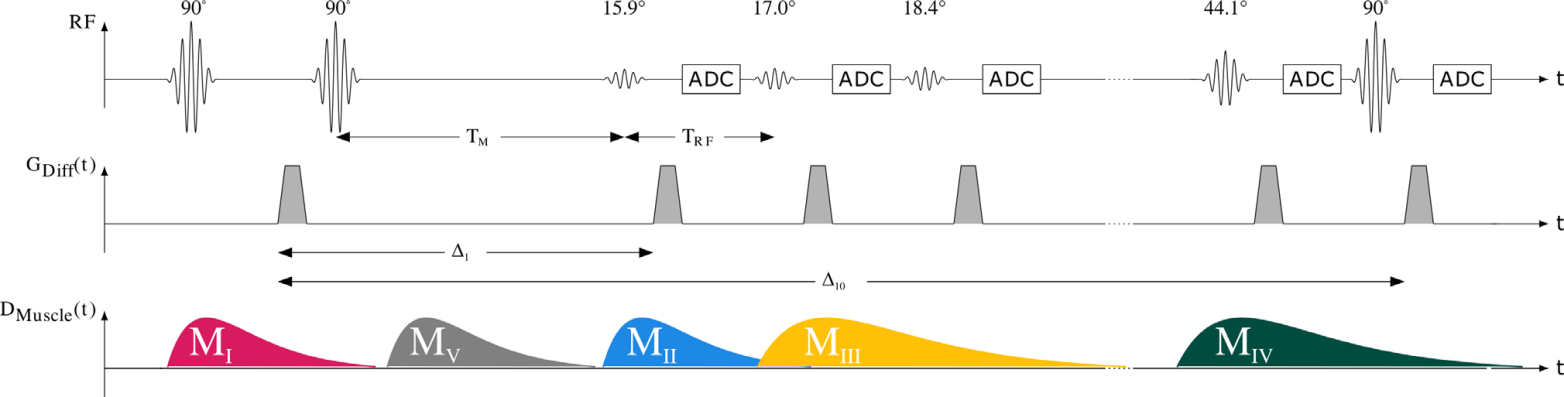
Asynchronous onset of muscular motion in relation to the multiple‐point diffusion‐weighted stimulated echo imaging sequence (MP‐DW‐STE) imaging sequence leads to five different kind of spontaneous mechanical activities of musculature (SMAM) motion pattern (M_I_ to M_V_) in the diffusion‐weighted images (DWI) time‐series. Investigation of dynamic characterization is only possible for the subgroup M_III_ where muscular motion starts after the first image and ends before the last image. Group M_V_ is not visible in the DWI time‐series because motion starts and relaxes between motion dephasing and rephasing gradients.

The contractile characteristics of the human skeletal muscular system can be divided into two temporal sections: a contraction phase and a relaxation phase. The contractile characteristics seem to be well represented by the course of the CSA of the SMAM in the successive images. Most of SMAMs that are visible in more than two subsequent DWI images have shown an increase of CSA peaking a maximum area and subsequent decrease. An example of muscular relaxation is demonstrated after the 6th image in the series shown in Figure 3. Therefore, it can be concluded that the MP‐DW‐STE sequence is able to image the contractile characteristics of SMAMs. For the different measurement settings I to III, the activation patterns were quite similar regarding active muscular regions, but the rate of activations showed significant differences. The b‐value of DW MRI sequences is mainly dependent on the gradient amplitude G_D_, the gradient duration δ, and the motion‐sensitizing time Δ. Because the b‐value was defined on the first image, the gradient amplitude is fixed for all other dephasing and rephasing motion‐sensitizing gradients within one measurement setting but changes between the measurement settings I to IV: G_D,**I**_ = 27 mT/m, G_D,**II**_ = 13.5 mT/m, G_D,**III**_ = 11.9 mT/m, and G_D,**IV**_ = 19.0 mT/m. For this, a relative motion difference of 87 μm (I), 173 μm (II), 198 μm (III), and 123 μm (IV) between the dephasing and rephasing motion‐sensitizing gradients results in a 180° phase shift. Therefore, the sensitivity of the MP‐DW‐STE sequence to visualize small muscular contractions is increased for I and decreased for II and III in relation to the standard STE sequence (IV). Based on this, the difference in the rate of activation between I (14.1% ± 8.1%) and III (11.9% ± 4.3%) in pECMs might be reflected by these changes of sensitivity. Furthermore, the increase in CSA for experimental setting I could also be a consequence. A dependence of the measured duration of SMAMs in the image series on the time of onset was shown for sequence settings I to III. The temporal sensitivity of a DW sequence, which is the probability to visualize SMAMs, is highly related to the timing of the sequence (i.e., mixing time interval T_M_).^41^ Because the muscular contractions occur asynchronous to the sampling pattern of the DW sequence, the timing of the onset of the muscular contraction during the imaging sequence in combination with the duration leads to certain likelihood that this movement can be visualized.^5^, ^41^ For example, SMAMs that start and end during the mixing interval T_M_ cannot be visualized by a DW sequence. The different types of possible configurations of muscular contraction that leads to different visible outcomes are depicted schematically in Figure 8 with corresponding class‐distribution scheme in Figure S4. Five different “types” of muscular contraction can be distinguished.

- M_I_: muscular contraction starts before the dephasing gradient in the initial phase of the MP‐DW‐STE sequence and relaxes during the mixing time interval T_M_ (i.e., relaxation before the first rephasing gradient) might lead to a phase shift for all imaging points in time and, therefore, a signal void in all following imaging cycles. The share of this type of SMAM is increased in II and III (25.3% and 25.1% in contrast to 15.2% [I]) because of the prolonged mixing time interval T_M_.
- M_II_: for M_II_, the muscular contraction is active after the dephasing gradient but before the rephasing gradient of the first image and ends during the imaging cycles. For this, the contraction time SMAM_TC_ cannot be determined, hence, the starting point is not known. An increase of this type of SMAM was found for II (17.6%) and III (20.5%) in contrast to I (5.1%). This difference is caused by the prolonged T_M_ in II and III in which the muscular contraction can evolve and start relaxation.
- M_III_: the muscular contraction starts after recording the first image (i.e., no visible SMAM in the first DWI) and ends before the last image in the cycle is acquired. In this case, the contraction and relaxation phase is visualized and reflected by increasing and decreasing CSA. II and III showed an increased relative amount of these contractions mainly because of the differences in motion sensitivity.
- M_IV_: muscular contraction shows an onset during the imaging cycles, but without finalized relaxation before recording the last image. Therefore, the contractile behavior cannot fully be determined.
- M_V_: complete contraction and relaxation within the mixing time interval T_M_ or after recording the last image does not lead to visualized SMAMs in DWI.^5^

Considering the time period between two consecutive RF excitations (i.e., T_RF_ = 40 ms) and the temporal period between first onset and maximum CSA (i.e., SMAM_TC_) the median contraction time for the soleus muscle was calculated to be 80 ms (95% CI: [80, 120 ms]) in (I) and 40 ms (95% CI: [40, 80 ms]) for (II) and (III). In contrast, the gastrocnemius medialis muscle showed a median contraction time of 40 ms (95% CI: [40, 80 ms]) to 80 ms (95% CI: [40, 80 ms]). Muscle fiber types can be mainly subdivided in slow twitch (fiber type I) and fast twitch (fiber type IIa/b/d/m).^1^ The soleus muscle is predominantly composed by slow twitch fibers (type I)^42^ with reported slow contraction times of 127 ± 6 ms (range, 64–251 ms)^43^ to 156.5 ± 14.7 ms^44^. In comparison, the contraction time of the gastrocnemius medialis muscle was reported to be 113.7 ± 19.6 ms^44^. A rather large deviation of those values given in the literature to the measured SMAM_TC_ is evident. This might be caused by the coarse temporal resolution of the MP‐DW‐STE in conjunction with the sensitivity of the sequence to visualize muscular motion depending on the amplitude of motion‐sensitizing gradients. For example, a relative motion difference of 87 μm leads to a phase shift of 180° for measurement setting I. Therefore, temporal regions with only a small motion difference, especially in the beginning of the muscular contraction or the end of muscular relaxation, might not be visualized by the sequence and the muscular contractions appears shorter than expected.

A median duration of SMAMs of 120 ms was found for soleus muscle and 120 to 160 ms for gastrocnemius muscle. From muscle ultrasound examinations, it was reported that fasciculations have shown a duration of 200 to 500 ms.^45^, ^46^ It can be derived from this study that MP‐DW‐STE imaging might underestimate the duration of fasciculations. This finding is likely to be related to the lower sensitivity of the MR sequence to very small displacements in musculature in comparison to ultrasound measurements. Another aspect could also be the number of images within the DWI time‐series because the measurements showed that 73.1% (I) of the SMAMs are not fully relaxed at the end of acquisition. For a more precise assessment of the SMAM duration, the RF time interval or the number of recordings during the DWI time‐series must be increased. Dynamic muscle ultrasound provides information about duration of the fasciculation^47^ because of the rather high temporal resolution and motion sensitivity (resolving changes in contractile length as low as 5 μm^48^); however, the pick‐up area is restricted to a few centimeters in this technique.^49^ Here, the visualization of fasciculations with DW‐MRI seems advantageous because of the large FOV without limitations in the transverse plane. This allows the simultaneous visualization of a larger amount of motor units in comparison to muscle ultrasound and enables imaging of the contraction time of fasciculation in deep muscles. Furthermore, assessment of fasciculating motor units in all muscular regions of the body is feasible enabling whole‐body imaging of fasciculation with determination of the rate and contraction time.

One limitation of this study is the small number of subjects some of whom have shown rather low frequency of SMAMs. However, the large number of repetitions per subject leads to sufficient data for the characterization of dynamic muscular contraction by MP‐DW‐STE sequences. Furthermore, preliminary investigations using a newly designed incoherent motion phantom underline the viability of this study. The presented sequence implementation is limited compared to nerve stimulation techniques^11^, ^13^ because of the relatively long time period between two RF excitations leading to only a coarse temporal resolution of SMAMs. For this, the onset and maximum CSA of the SMAM cannot be optimally estimated. Additionally, very short spontaneous muscular contractions may not be adequately visualized because of the limited temporal resolution of the MR sequence. In addition, the amplitude of the motion‐sensitizing gradients has a large impact on the measurements in terms of number of SMAMs and duration. However, no additional equipment is required to carry out fully non‐invasive MR examinations (without external nerve stimulation). Measurements were only conducted with the incoherent motion phantom in one specific direction (i.e., within the axial plane) and with a slow‐moving motion bead. Further studies evaluating the sensitivity of the MR sequence regarding frequency and duration of induced movements, as well as, the dependency of the angle to the imaging plane might be of interest. Therefore, an incoherent motion phantom with a more precise control allowing very small and fast movements has to be built. Evaluation was restricted on the two muscles soleus und gastrocnemius medialis because of the expected low activities in other muscular regions. However, further work in this field of research should also consider other muscles, like tibialis anterior, because spontaneous activities might be relevant especially in patient groups. Therefore, a study with patient groups potentially showing a distinct change in the activation pattern of motor units and the mechanical response is considered a reasonable next step further. In comparison to clinically established needle electromyography for the detection of spontaneous activity with a high temporal resolution of the myoelectric signal, this MR technique enables to visualize the temporal course of mechanical activities in a larger FOV. However, needle electromyography is much less expensive and established in the clinical setting. Clinical translation of the proposed MR technique seems possible, but its value is still to be determined.

## 5 CONCLUSION

The systematic MR studies on a fully controllable incoherent motion phantom as well as in vivo measurements in healthy subjects demonstrate that the acquisition of several points in time of the human spontaneous muscular twitch contraction (SMAM) using a multiple‐point in time DW‐STE MRI sequence is feasible. In addition, no external myoelectric stimulation is required to record the temporal course of a single spontaneous muscle twitch contraction. For imaging of the contraction time, large amplitude motion‐sensitivity gradients might be optimal with short temporal period between RF excitations. Faster sampling of k‐space is required to shorten the interval between two RF excitations allowing more sampling points within the acquisition window. However, this goes along with reduced spatial resolution and overall image quality. Furthermore, triggering the imaging sequence by recordable myoelectric signals might be challenging, but would improve the understanding of spontaneous muscular contraction in the human skeletal muscular system visible in MRI.

## CONFLICT OF INTEREST STATEMENT

Nothing to declare.

## Supporting information


**Table S1.** (A) Median number of images indicating SMAMs per intra‐series imaging point in time for all three measurement settings. Differences between all three measurement settings (*p* = 0.008, Friedman test) as well as between the 10 imaging time points for each measurement (*P*_adj_ ≤0.001, Wilcoxon test) were significant. Pair‐wise test: †‡ (*P*_adj_ <0.05, Wilcoxon test). (B) Median cross‐sectional areas of SMAMs in the unit of voxels for all three measurement settings. A significant difference was revealed for testing all three measurements (*p* = 0.006, Friedman test). (α: *P*_adj_ = 0.012, Wilcoxon test, β: *P*_adj_ = 0.011, Wilcoxon test).
**Figure S1.** (A) Photography of the disassembled incoherent motion phantom. (B) Schematic axial view of the incoherent motion phantom with two intra‐slice and four inter‐slice tubes. Intra‐slice tubes filled with water and cellulose (2%) were utilized for inducing incoherent motion with a motion bead (red) connected to thin filaments. An additional layer of rubber was inserted at the end of the intra‐slice tubes to reduce the exchange between surrounding water. Four inter‐slice tubes were filled with a soy‐lecithin solution for adjusting T_1_‐values mimicking relaxation times of different tissues enabling investigations regarding T_1_‐dependent signal intensity variation.
**Figure S2.** Overall percentage Event Count Maps (pECMs) visualized for all seven subjects and all four MR settings. The spatial distribution of the activation patterns within subjects remains similar using different sequence settings.
**Figure S3.** Exemplary calculation of the relative proportion of the individual subgroups of SMAMs for class: (A), (B), (C), (D) and (E).
**Figure S4.** Four different classes of visible SMAMs categorized by onset and duration in the course of the MR sequence. SMAMs of the 5th (M_V_) class are not visible in DWI because contraction and relaxation is not during the motion‐sensitive period of the MR sequence and therefore the relative amount cannot be depicted.
