# Supplementary material for: The dynamic course of spontaneous muscular contractions assessed using multiple‐point acquisition in diffusion‐weighted stimulated echo imaging
Source: Magn Reson Med. 2025 Jun 18;95(1):517–30. doi: 10.1002/mrm.30576 (PMC12620152; doi:10.1002/mrm.30576)
Supplement: Supplementary file 1 — Table S1. (A) Median number of images indicating SMAMs per intra‐series imaging point in time for all three measurement settings. Differences between all three measurement settings (p = 0.008, Friedman test) as well as between the 10 imaging time points for each measurement (P adj ≤0.001, Wilcoxon test) were significant. Pair‐wise test: †‡ (P adj <0.05, Wilcoxon test). (B) Median cross‐sectional areas of SMAMs in the unit of voxels for all three measurement settings. A significant difference was revealed for testing all three measurements (p = 0.006, Friedman test). (α: P adj = 0.012, Wilcoxon test, β: P adj = 0.011, Wilcoxon test). Figure S1. (A) Photography of the disassembled incoherent motion phantom. (B) Schematic axial view of the incoherent motion phantom with two intra‐slice and four inter‐slice tubes. Intra‐slice tubes filled with water and cellulose (2%) were utilized for inducing incoherent motion with a motion bead (red) connected to thin filaments. An additional layer of rubber was inserted at the end of the intra‐slice tubes to reduce the exchange between surrounding water. Four inter‐slice tubes were filled with a soy‐lecithin solution for adjusting T1‐values mimicking relaxation times of different tissues enabling investigations regarding T1‐dependent signal intensity variation. Figure S2. Overall percentage Event Count Maps (pECMs) visualized for all seven subjects and all four MR settings. The spatial distribution of the activation patterns within subjects remains similar using different sequence settings. Figure S3. Exemplary calculation of the relative proportion of the individual subgroups of SMAMs for class: (A), (B), (C), (D) and (E). Figure S4. Four different classes of visible SMAMs categorized by onset and duration in the course of the MR sequence. SMAMs of the 5th (MV) class are not visible in DWI because contraction and relaxation is not during the motion‐sensitive period of the MR sequence and therefore the relative amount cannot be depi [file MRM-95-517-s001.docx]

**Supporting Information**

**Supporting Table S1: A:** Median number of images indicating SMAMs per intra-series imaging point in time for all three measurement settings. Differences between all three measurement settings (P = 0.008, Friedman test) as well as between the ten imaging time points for each measurement (P_adj_ ≤ 0.001, Wilcoxon test) were significant. Pair-wise test: †‡ (P_adj_ < 0.05, Wilcoxon test). **B:** Median cross-sectional areas of SMAMs in the unit of voxels for all three measurement settings. A significant difference was revealed for testing all three measurements (P = 0.006, Friedman test). (α: P_adj_ = 0.012, Wilcoxon test, β: P_adj_ = 0.011, Wilcoxon test).

| **A** | **Number of images indicating SMAMs per intra-series imaging point in time** | | | | | | | | | |
| --- | --- | --- | --- | --- | --- | --- | --- | --- | --- | --- |
|  | **1^st^** | **2^nd^** | **3^rd^** | **4^th^** | **5^th^** | **6^th^** | **7^th^** | **8^th^** | **9^th^** | **10^th^** |
| **I** | 9 †  (2, 12) | 17 ‡  (2, 21) | 22  (4, 28) | 28  (4, 32) | 35  (3, 40) | 36  (3, 46) | 39 †  (3, 44) | 40 †‡  (3, 48) | 44 †‡  (4, 47) | 38 †‡  (4, 44) |
| **II** | 17 †  (6, 28) | 18  (7, 30) | 20  (9, 35) | 22 †  (10, 39) | 26 †  (12, 36) | 27 †  (11, 37) | 27  (9, 36) | 26  (9, 38) | 25 †  (9, 38) | 21  (9, 38) |
| **III** | 17 †  (7, 21) | 19  (10, 23) | 19  (9, 24) | 20  (10, 28) | 23  (10, 29) | 21  (10, 30) | 21  (10, 30) | 23 †  (11, 29) | 22  (9, 26) | 21  (9, 27) |
|  |  |  |  |  |  |  |  |  |  |  |
| **B** | **Cross-sectional areas of SMAMs in the unit of voxels** | | | | | | | | | |
|  | **1^st^** | **2^nd^** | **3^rd^** | **4^th^** | **5^th^** | **6^th^** | **7^th^** | **8^th^** | **9^th^** | **10^th^** |
| **I**^αβ^ | 10  (8, 14) | 15  (10, 17) | 17  (15, 19) | 18  (16, 22) | 19  (17, 23) | 21  (19, 24) | 22  (19, 26) | 21  (19, 26) | 21  (19, 24) | 21  (18, 24) |
| **II**^α^ | 13  (11, 16) | 14  (11, 16) | 15  (13, 17) | 14  (12, 16) | 13  (12, 16) | 14  (12, 16) | 15  (13, 17) | 14  (12, 17) | 14  (12, 16) | 15  (12, 18) |
| **III**^β^ | 12  (10, 14) | 14  (11, 16) | 15  (13, 17) | 15  (12, 17) | 14  (12, 16) | 15  (14, 17) | 14  (10, 15) | 12  (10, 14) | 13  (11, 14) | 10  (8, 12) |


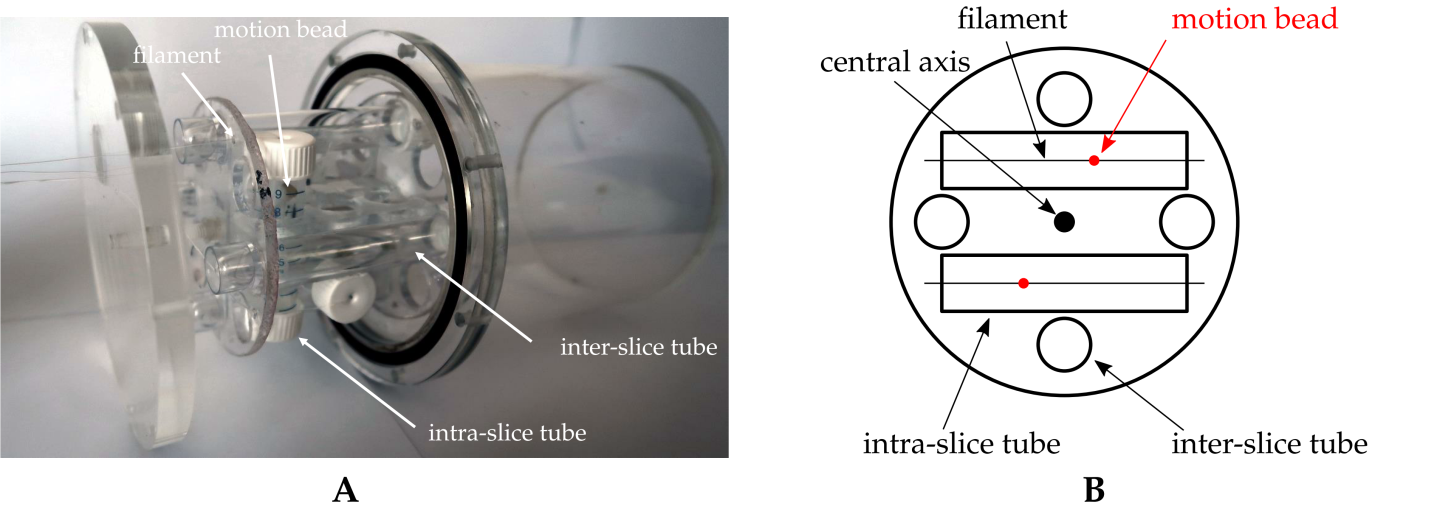


**Supporting Figure S1: A:** Photography of the disassembled incoherent motion phantom. **B:** Schematic axial view of the incoherent motion phantom with two intra-slice and four inter-slice tubes. Intra-slice tubes filled with water and cellulose (2%) were utilized for inducing incoherent motion with a motion bead (red) connected to thin filaments. An additional layer of rubber was inserted at the end of the intra-slice tubes to reduce the exchange between surrounding water. Four inter-slice tubes were filled with a soy-lecithin solution for adjusting T_1_-values mimicking relaxation times of different tissues enabling investigations regarding T_1_-dependent signal intensity variation.


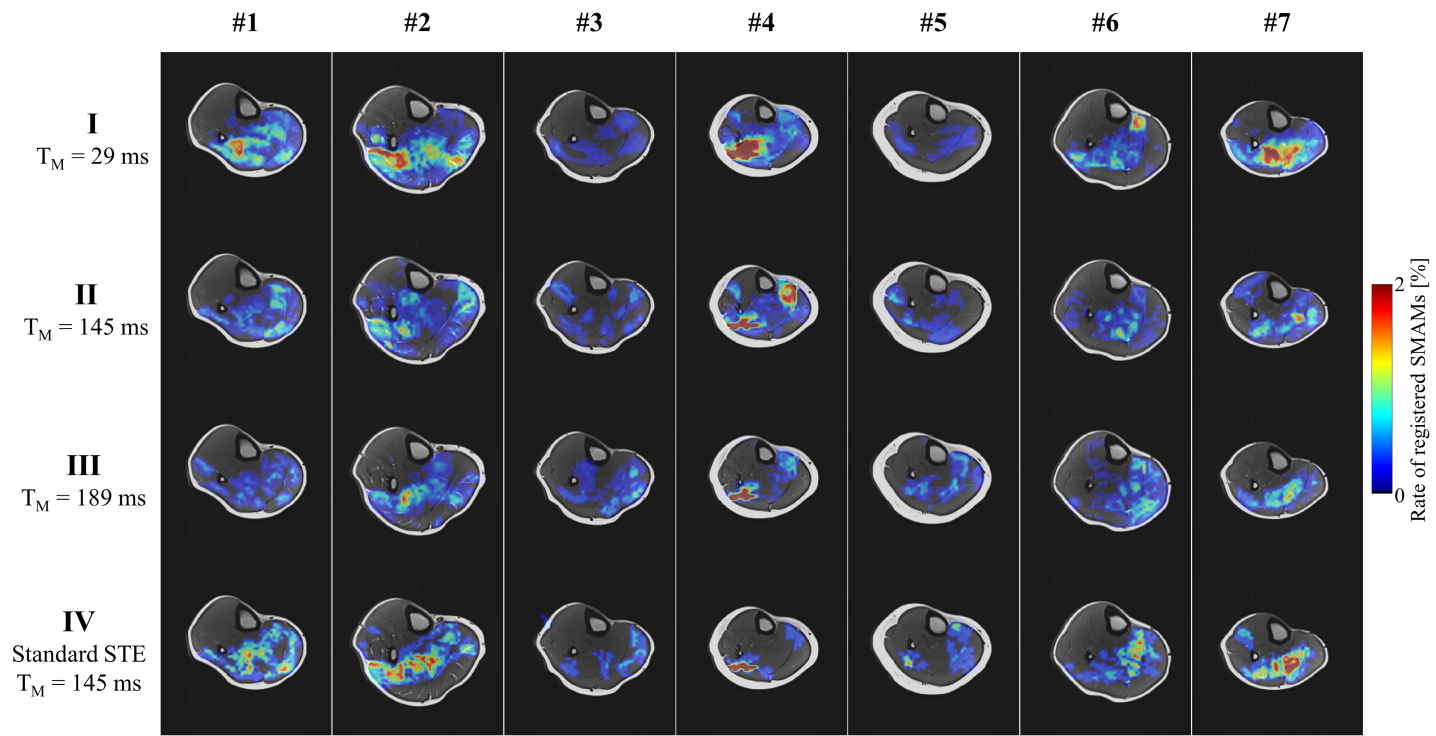


**Supporting Figure S2:** Overall percentage Event Count Maps (pECMs) visualized for all seven subjects and all four MR settings. The spatial distribution of the activation patterns within subjects remains similar using different sequence settings.

**A:** Calculations for setting

**I:** 1$5.2\%$

**II:** $25.3\%$

**III:** $25.1\%$

**B:** Calculations for setting

**I:** $0.4\%+4\cdot0.8\%+1.5\%=5.1\%$

**II:** $0.4\%+1.2\%+3\cdot1.6\%+2.3\%+3.5\% +5.4=17.6\%$

**III:** $0.9\%+1.4\%+1.8\%+2\cdot2.7\%+2\cdot3.2\%+4.6\%=20.5\%$

**C:** Calculations for setting

**I:** $15.2\%+10.6\%+9.5\%+5.7\%+8.3\%+5.3\%+6.4\%+3.4\%+8.7\%=73.1\%$

**II:** $25.3\%+4.3\%+3.5\%+1.9\%+2.3\%+4.7\%+1.9\%+6.2\%+5.8\%=55.9\%$

**III:** $25.1\%+1.8\%+2.7\%+1.4\%+2.7\%+1.8\%+5.9\%+6.4\%+5.9\%=53.7\%$

**D:** Calculations for setting

**I:** $8\cdot0.4\%+5\cdot0.8\%+4\cdot1.1\%+7\cdot1.5\%=22.1\%$

**II:** $5\cdot0.4\%+6\cdot0.8\%+6\cdot1.2\%+4\cdot1.6\%+2\cdot1.9\%+2.7\%=26.9\%$

**III:** $6\cdot0.5\%+6\cdot0.9\%+4\cdot1.4\%+4\cdot1.8\%+2\cdot2.3\%=25.8\%$

**E:** Calculations for setting

**I:** $2\cdot0.4\%+0.8\%+1.1\%+1.5\%=4.2\%$

**II:** $3\cdot0.4\%+2\cdot0.8\%+1.2\%+3.5\%=7.5\%$

**III:** $2\cdot0.5\%+3.2\%=4.2\%$

**Supporting Figure S3:** Exemplary calculation of the relative proportion of the individual subgroups of SMAMs for class: A), B), C), D) and E).


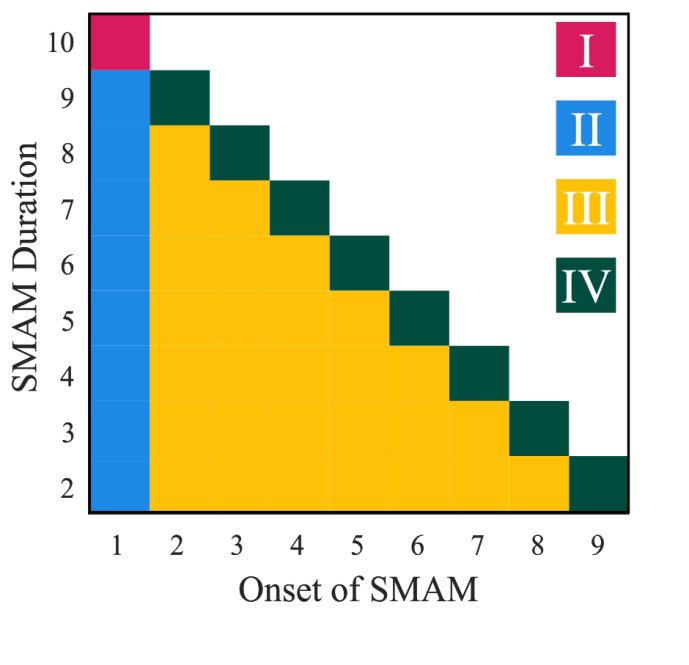


**Supporting Figure S4:** Four different classes of visible SMAMs categorized by onset and duration in the course of the MR sequence. SMAMs of the 5^th^ (M_V_) class are not visible in DWI because contraction and relaxation is not during the motion-sensitive period of the MR sequence and therefore the relative amount cannot be depicted.
